# Molecular Evolution of Hemagglutinin (*H*) Gene in Measles Virus Genotypes D3, D5, D9, and H1

**DOI:** 10.1371/journal.pone.0050660

**Published:** 2012-11-29

**Authors:** Mika Saitoh, Makoto Takeda, Koichi Gotoh, Fumihiko Takeuchi, Tsuyoshi Sekizuka, Makoto Kuroda, Katsumi Mizuta, Akihide Ryo, Ryota Tanaka, Haruyuki Ishii, Hayato Takada, Kunihisa Kozawa, Ayako Yoshida, Masahiro Noda, Nobuhiko Okabe, Hirokazu Kimura

**Affiliations:** 1 Gunma Prefectural Institute of Public Health and Environmental Sciences, Maebashi-shi, Gunma, Japan; 2 Department of Virology III, National Institute of Infectious Diseases, Musashimurayama-shi, Tokyo, Japan; 3 Pathogen Genomics Center, National Institute of Infectious Diseases, Shinjuku-ku, Tokyo, Japan; 4 Yamagata Prefectural Institute of Public Health, Yamagata-shi, Yamagata, Japan; 5 Department of Molecular Biodefence Research, Yokohama City University Graduate School of Medicine, Yokohama-shi, Kanagawa, Japan; 6 Department of Surgery, Institute of Medical Sciences, Kyorin University, Mitaka-shi, Tokyo, Japan; 7 Department of Respiratory Medicine, Kyorin University, School of Medicine, Mitaka-shi, Tokyo, Japan; 8 Aomori Prefectural Institute of Public Health and Environment, Aomori-shi, Aomori, Japan; 9 Infectious Disease Surveillance Center, National Institute of Infectious Diseases, Musashimurayama-shi, Tokyo, Japan; Columbia University, United States of America

## Abstract

We studied the molecular evolution of *H* gene in four prevalent Asian genotypes (D3, D5, D9, and H1) of measles virus (MeV). We estimated the evolutionary time scale of the gene by the Bayesian Markov Chain Monte Carlo (MCMC) method. In addition, we predicted the changes in structure of H protein due to selective pressures. The phylogenetic tree showed that the first division of these genotypes occurred around 1931, and further division of each type in the 1960–1970s resulted in four genotypes. The rate of molecular evolution was relatively slow (5.57×10^−4^ substitutions per site per year). Only two positively selected sites (F476L and Q575K) were identified in H protein, although these substitutions might not have imparted significant changes to the structure of the protein or the epitopes for phylactic antibodies. The results suggested that the prevalent Asian MeV genotypes were generated over approximately 30–40 years and H protein was well conserved.

## Introduction

Measles virus (MeV) of genus *Morbillivirus* and family *Paramyxoviridae* causes acute and highly contagious measles infection in humans [1], [2]. Since the year 2000, the number of patients in many countries with measles has continued to decrease due to widespread measles immunization programs [3]. Nevertheless, an estimated 300,000 cases were reported and 140,000 young children died from measles globally in 2010 (Media Centre. Measles. World Health Organization. http://www.who.int/mediacentre/factsheets/fs286/en/index.html). Thus, the World Health Organization has focused on the infection as an eliminative disease.

Many genotypes of MeV have been identified, although the virus may be confirmed as a monoserotype [1], [2]. Interestingly, there are associations between the prevalence of each genotype and geographical area [4]. For example, genotypes D4 and D6 are mainly detected in European countries, while genotypes D3, D5, D9, and H1 are mainly detected in Asian countries [4]. At present, in some countries that have eliminated measles, a small numbers of cases are caused by a number of different genotypes that reflect various sources of imported viruses [2]. The MeV genome encodes some essential structural proteins such as hemagglutinin (H) and fusion (F) proteins [1]. The H protein generally regulates viral adsorption and entry, and that of the vaccine strains shows hemagglutinin activity as well [1]. The neutralizing antibodies against H protein act as protective antibodies in MeV infection. In addition, recent studies have shown the detailed structure of the H protein and the epitopes for the neutralizing antibodies [5], [6]. Such antigenic changes may occur through positively selected amino acid substitutions due to selection pressures in the host. Indeed, attachment glycoprotein (G protein), an essential antigen of respiratory syncytial virus, shows frequent positive selections in the epitopes of the protein, whereas no positive selection sites have been found in the HN protein (a major antigen) of human parainfluenza virus type 1 [7], [8]. This suggests that the frequency of the positive selection sites differs among the major antigens of these viruses, even though the viruses all belong to the same family, *Paramyxoviridae*. Thus, it may be important to analyze the molecular evolution of *H* gene in MeV.

The Bayesian Markov Chain Monte Carlo (MCMC) method enables the evolutionary time scale to be estimated [9], [10]. Furthermore, detailed changes in H protein structure may be predictive. In the present study we conducted a detailed genetic analysis of the gene and predicted changes in the structure of H protein to gain a better understanding of the evolution of *H* gene in prevalent Asian MeV genotypes (D3, D5, D9, and H1).

## Results

### Phylogenetic Analysis Using the Bayesian MCMC Method on the *H* Coding Region of MeV

**Figure 1:**
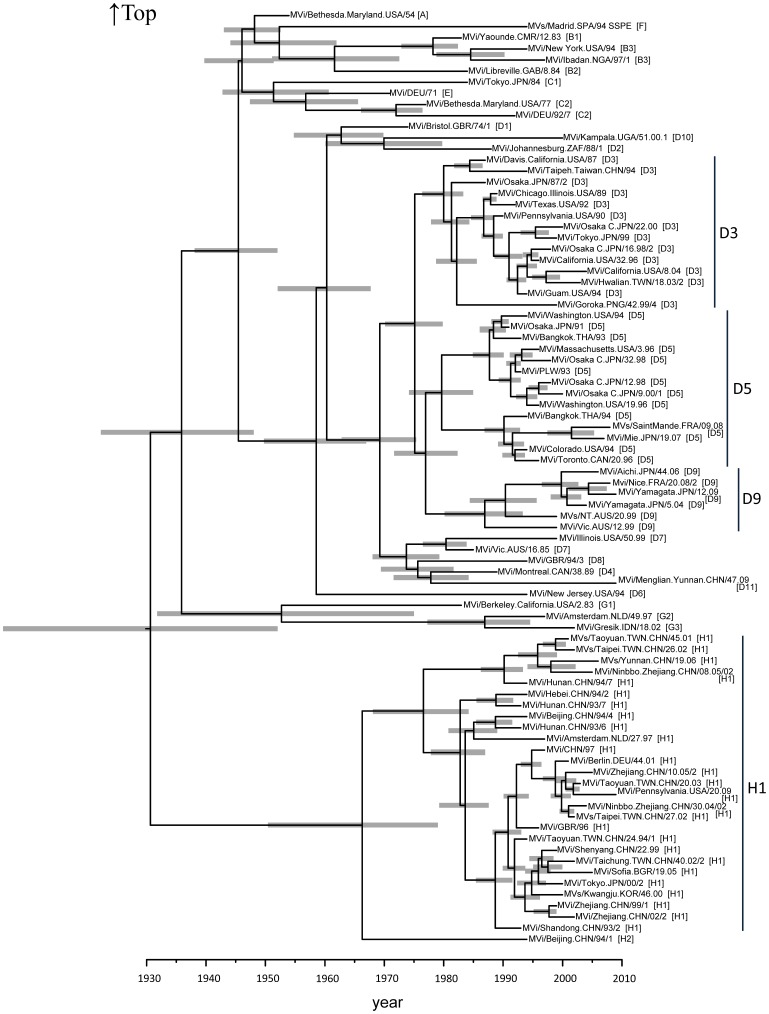
Phylogenetic tree of *H* gene by Bayesian Markov Chain Monte Carlo (MCMC) method. The MCMC tree was based on the full nucleotide sequence of *H* gene (1854 nt) visualized in FigTree. The branch length reflects the evolutionary rate of individual sequences and their reconstructed ancestors. Gray bars indicate 95% confidence intervals for the estimated year. MeV strains were named according to WHO standard nomenclature. The strain names provide following information. MVi: sequence derived from RNA extracted from measles virus isolate in cell culture, MVs: sequence derived from RNA extracted from clinical material/city or province and country: use ISO-3 letter designation/date of onset of rash by epidemiological week and year, and isolate or sequence numbers/genotype in square brackets.

The phylogenetic tree constructed using the Bayesian MCMC method with the nucleotide sequences of the *H* gene (1854 nt) for various genotypes (A to H) of MeV is shown in Fig. 1. The year of the first major division in the present tree was estimated as approximately 1931 (95% confidence interval [CI] 1906–1952). The D3, D5, and D9 subdivisions occurred in approximately 1975 (95% CI 1970–1980) and 1977 (95% CI 1972–1982), and the H1 and H2 subdivisions occurred in approximately 1966 (95% CI 1950–1979), resulting in the formation of 4 genotype clusters [D3 (14 strains), D5 (14 strains), D9 (6 strains), and H1 (27 strains)]. Further division of each genotype occurred in approximately 1980 (95% CI 1976–1983) for D3, 1980 (95% CI 1974–1985) for D5, 1987 (95% CI 1980–1993) for D9, and 1977 (95% CI 1968–1984) for H1. The CIs for each node of the phylogenetic tree are expressed as gray bars in Fig. 1. The rate of molecular evolution was estimated from the tree as 5.57×10^−4^ substitutions per site per year (95% CI 4.50×10^−4^–6.81×10^−4^).

### Analysis of Selective Pressure of *H* Gene in MeV

Selection pressure analysis was performed in the strains of the various genotypes (A to H) in MeV *H* gene, and dN/dS values were calculated by the single likelihood ancestor counting (SLAC), fixed effects likelihood (FEL), and internal fixed effects likelihood (IFEL) methods, significant at the *p*<0.05 level. The global estimate of dN/dS was 0.22 (95% CIs of 0.19–0.24) by SLAC. Estimates of the dN/dS ratio of two codons were detected and two amino acid substitutions were estimated (F476L and Q575K) by the FEL and IFEL methods (Table 1). Negatively selected sites were detected by each of the three methods and 28 codons were identified (Table 1).

**Table 1:** Positive and negative selection sites in MeV H coding region in the present study.

Positive selection sites.			
aa position	Change	†FEL	††IFEL
220V	T or I		*
282N	K or H or D		*
235E and G	G or E or A	*	
285S	G or N		*
451V	E or M or A		*
476F	L	*	*
546S	G	*	
575Q	K	*	*
**Negative selection sites**			
**aa common position**		† **FEL**	†† **IFEL**
3P,7R,13K,14D,16P,90D,209Y,228Y,237P,242K,259V,297A,374D,388G,400A,401P,487I,489E,492E,515V,538V,541Y,548S,563P,588S,606C,611E,612D		131sites	28sites

*p*<0.05.

† FEL: Fixed effects likelihood.

†† IFEL: International fixed effects likelihood.

### Location of the Two Positively Selected Amino Acid Sites

**Figure 2:**
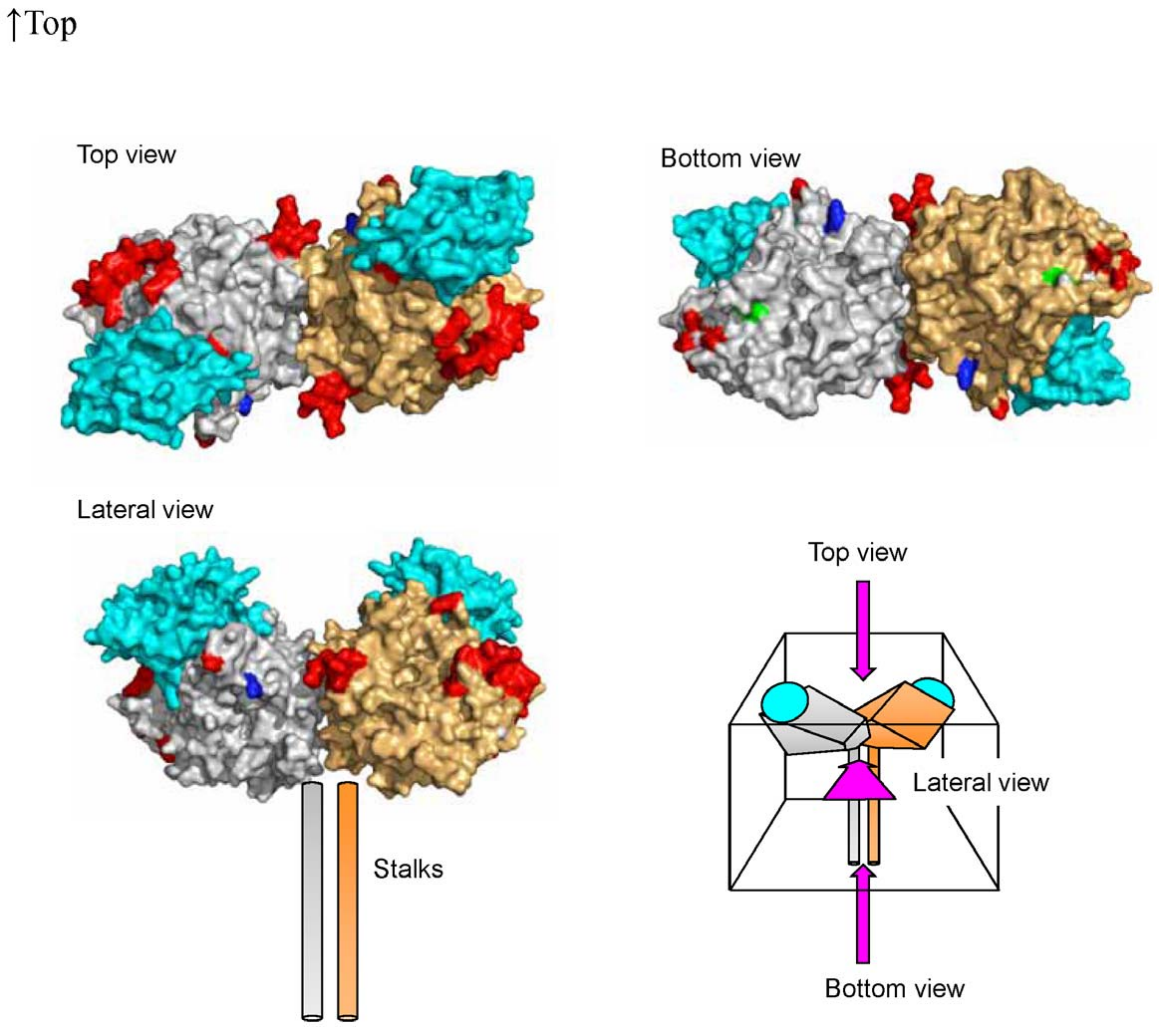
The predicted structure of H protein in MeV. The H head dimers are shown from the top, bottom, and lateral angles. Each monomer is shown in gray or orange. Residues reported to constitute epitopes are shown in red. SLAM is shown in cyan. Positively selected amino acid sites, 476 and 575, are shown in light green and blue, respectively.

The two amino acid positions (476 and 575) were shown on the H protein structure [6]. The residues are exposed on the surface (light green and blue, respectively; Fig. 2). Therefore, they may be parts of epitopes, but to date there are no reports showing that the regions containing residues 476 or 575 constitute epitopes (known epitopes are shown in red in Fig. 2). The structural data showed that these residues are located at the bottom and lateral surfaces, respectively, of the H head dimer, and distal from the SLAM-binding site (SLAM is shown in cyan in Fig. 2).

## Discussion

We analyzed the molecular evolution of *H* gene in genotypes D3, D5, D9, and H1 of MeV, which are prevalent in Asian countries including China and Japan. First, we estimated the evolutionary time scale of the gene using the Bayesian MCMC method. The first division of these genotypes in the present tree was estimated as approximately 1931, and each genotype further divided in the 1960–70s, resulting in 4 genotypes. In addition, only two positively selected sites were observed, although these changes might not reflect significant structural changes in H protein. The results suggested that the 4 genotypes were formed over a period of approximately 30–40 years (from approximately 1931 to the 1960–70s) and the structure of the H protein has been well conserved.

MeV genotype D3 was mainly detected in various Western Pacific countries including Japan, Australia, Papua New Guinea, and the Philippines during 1983–2006, and was endemic in Papua New Guinea and possibly the Philippines from 2002–2006 [2], [11], [12]. The first major division of type D3 was estimated at around 1975, and the ancestral strains further subdivided from around 1980 (Fig. 1). Genotype D5 has been prevalent in Japan, Australia, and Cambodia since 1985 [2], [11], [12]. Until 2001, both D3 and the D5 genotypes were endemic in Japan. The first major division of type D5 was estimated at around 1975, and extensive branching into two clusters occurred around 1980. Furthermore, it is suggested that the ancestral strains have undergone further divisions from around 1990. Genotype D9 was first described after importation to Australia from Indonesia in 1999 and was associated with an outbreak in Japan in 2004 [2], [11], [12]. The strains detected in Japan and France in the mid-2000s had diverged in approximately 1990 from the strains detected in Australia in 1999. Genotype H1, detected during the large measles epidemic in Korea in 2000–2001, has been prevalent in China, Japan, Korea, Vietnam, and Australia since 1993 and is mainly associated with transmission within China or in importations from China [2], [11], [12]. This genotype diverged into plural clusters after 1977. The Korean strain detected in Korea in 2000 evolved from one Chinese strain clusters in about 1995. The results showed that the Asian prevalent MeV genotypes D3, D5, D9, and H1 were generated over approximately 30–40 years, and the ages of the diverged clusters for each genotype could be predicted. In the present study, it is estimated that each genotype, e.g., D3, D5, D9, and H1, was generated from the 1960s to 70s. However, there was a difference in the year that the viruses were generated according to the phylogenetic tree based on the Bayesian MCMC method and the year that they were first detected. For example, the D9 strain was shown to be generated approximately 40 years ago using the phylogenetic tree, while the virus was detected in 1999. This has been seen in other viruses, such as rubella virus [13]. Indeed, Zhu et al. showed that cluster 1 isolates of Chinese genotype 1E rubella virus were collected from 2001 to 2009, while the viruses were estimated to have appeared in 1997 according to the MCMC method. To solve this discrepancy, additional studies are needed.

We analyzed the rate of molecular evolution from the tree as 5.57×10^−4^ substitutions per site per year. The rates of evolution of *H* gene in seasonal influenza virus subtype A and *G* gene in respiratory syncytial virus are estimated at about 10^−3^ substitutions per site per year [14], [15]. In addition, we reported that the rate of *HN* gene in human parainfluenza virus type 1 is estimated at 7.68×10^−4^ substitutions/site/year [8]. These results suggest that the rate of evolution of *H* gene of MeV may be similar to the rate of *HN* gene of human parainfluenza virus type 1, and their rates of molecular evolution may be relatively slow [8].

We estimated positively and negatively selected sites in the gene by the SLAC, FEL, and IFEL methods. Positive selection shows a survival advantage under the selective constraints that confront the viral population [16]. Negative selection plays an important role in maintaining the long-term stability of biological structures by removing deleterious mutations [16], [17]. In this study, two positively selected sites (F476L and Q575K) were found. Woelk et al demonstrated 14 positively selected sites in 50 strains of *H* gene in all genotypes of MeV [18]. Of these, amino acid positions corresponding to 476 and 575 are compatible with our results. Region 463–477 including site 476 and region 561–575 including site 575 react with human sera. Furthermore, region 463–477 is a possible candidate for interaction with the CD46 receptor. In addition, Corey and Iorio have shown that amino acid substitutions at region 473–477 including 476 drastically reduce hemagglutinin activity associated with fusion promotion [19]. In this study, it is suggested that the amino acid substitutions of two positively selected sites share these particular abilities in MeV *H* gene. Twenty- eight negatively selected sites were found (Table 1). These sites may be optimized biological structures, thus further analysis of the biological properties of H glycoprotein in MeV is required. In addition, it may be important to make a distinction between the branched year of the viruses on a phylogenetic tree and the nucleotide and amino acid substitutions as positive selection. Although this question could not be elucidated in the present study, further studies regarding these relationships are needed.

Finally, we predicted the changes of the epitopes [5], [6] for neutralizing antibodies against H protein by substitutions of the amino acid due to positive selection pressure. The amino acid changes at positively selected sites may confer an advantage to MeV in terms of transmission. The residues at amino acid positions 476 and 575 are exposed on the surface, but are located distal from receptor binding site and unrelated to known neutralizing epitopes. Therefore, the amino acid substitutions at these positions may not significantly affect the efficacy of humoral immunity against MeV. These data suggested that in these several decades none of the amino acid substitutions on the epitopes succeeded to give MeV better fitness in nature significantly. These observations could provide a rationale for the high efficacy of currently used MeV vaccines against all MeV strains circulating. In conclusion, it suggested that the prevalent Asian MeV genotypes were generated over approximately 30–40 years and H protein was well conserved. As an essential molecule of these viruses, further analysis of the biological properties of H glycoprotein in MeV is required. Thus, additional and larger molecular epidemiological studies are required to give better understanding of the etiology of MeV.

## Materials and Methods

### Strains

We comprehensively collected total 162 strains of various genotypes of *H* gene sequence (1854 nt), such as 23 reference strains (genotypes A, B1 to B3, C1, C2, D1, D2, D4, D6 to D8, D10, D11, E, F, G1 to 3, and H2 [2]) and Asian-prevalent genotype strains, including the reference strains (D3, 37 strains; D5, 34 strains; D9. 11 strains; and H1, 57 strains), from MeaNS (http://www.hpa-bioinformatics.org.uk/Measles/Public/Web_Front/main.php) [20]. Their sequences correspond to positions 21–1874 (1854 nt) of MVi/Chicago.Illinois.USA/89 (reference strain for genotype D3) (GenBank accession number M81895). The MeV sequences were aligned using the ClustalW web server (http://www.ddbj.nig.ac.jp/index-j.html).

### Phylogenetic Analysis by the Bayesian MCMC Method

Using all of the present strains to estimate the rate of molecular evolution (and hence a time scale) and evolutionary relationships, phylogenetic analyses were performed using the Bayesian MCMC method in the BEAST program (version 1.7.2) [21]. The time of the most recent common ancestor (*t*MRCA) with a 95% highest posterior density (HPD) was estimated by Bayesian molecular dating as described previously [9], [10]. The MeV sequences were aligned using the ClustalW web server (http://www.ddbj.nig.ac.jp/index-j.html). We removed the identical sequences from within the MeV *H* coding region of the genotype strains. The dataset was analyzed under a lognormal relaxed uncorrelated clock using the general time reversible (GTR) model with the gamma distributed rates across sites (GTR+Γ) model selected by the KAKUSAN4 program (version 4.0). The MCMC chain was run for 30,000,000 steps and sampled every 1,000 steps. Uncertainty in the estimates was indicated by the 95% HPD intervals. The parameter outputs generated by the Bayesian MCMC runs and convergence on the basis of the effective sampling size after a 10% burn-in were analyzed using the TRACER program (version 1.5). The trees were summarized in a target tree using the Tree Annotator program (version 1.7.2) by choosing the tree with the maximum posterior probabilities after a 10% burn-in. The phylogenetic tree was viewed in FigTree (version 1.3.1; available at: http://beast.bio.ed.ac.uk). Next, to understand the phylogenetic tree easily, we selected and removed the sequences with high homogeneity and identical isolation years in each cluster of the tree. As a result, the present phylogenetic tree included 23 genotypes of the reference strains and the Asian-prevalent strains, including the reference strains (14 strains of D3, 14 strains of D5, 6 strains of D9, and 27 strains of H1).

### Selective Pressures Analysis

To evaluate selective pressures on the *H* coding regions among all MeV strains, the rates of synonymous (dS) and non-synonymous (dN) changes at amino acid sites were estimated by conservative SLAC, FEL, and IFEL methods using ML available on the Datamonkey webserver (http://www.datamonkey.org/) [22]. The SLAC method is suitable for fast likelihood-based “counting methods” that employ either a single most likely ancestral reconstruction, weighted across all possible ancestral reconstructions, or sampling from ancestral reconstructions. The FEL method directly estimates dN and dS substitution rates at each site. The IFEL method is tested for only along internal branches of the phylogeny in the same manner. To examine the dN and dS rates, these methods were performed incorporating the GTR model of nucleotide substitution and the phylogenetic tree deduced using the NJ method. The dN/dS values of each codon and branch of the present phylogenetic tree were assessed as described previously [23]. Positive (dN>dS) and negative (dN<dS) selections were predicted using the *p*-value [23].

### Prediction of Epitopes for Neutralizing Antibody Against MeV H Protein Based on the Deduced Amino Acid Substitutions

To clarify the location of substituted amino acids on the H protein, we mapped the positively and negatively selected sites as previously described [5], [6]. Figures were produced using PyMOL (DeLano Scientific LLC, Palo Alto, CA, USA. http://www.pymol.org.).
